# Targeting STAT3 in tumor-associated antigen-presenting cells as a strategy for kidney and bladder cancer immunotherapy

**DOI:** 10.3389/fimmu.2023.1274781

**Published:** 2024-01-08

**Authors:** Marice B. Alcantara, Wilson S. Tang, Dongfang Wang, Damian Kaniowski, Elaine Kang, Nazli Dizman, Alexander Chehrazi-Raffle, Luis Meza, Zeynep Zengin, Jeremy Hall, JoAnn Hsu, Colt Egelston, Dayson Moreira, Alan Horsager, Sumanta K. Pal, Marcin Kortylewski

**Affiliations:** ^1^ Department of Immuno-Oncology, Beckman Research Institute, City of Hope National Medical Centre, Duarte, CA, United States; ^2^ Department of Medical Oncology, City of Hope National Medical Centre, Duarte, CA, United States; ^3^ MD Anderson Cancer Center, Department of Hematology and Oncology, Houston, TX, United States; ^4^ Duet Biotherapeutics, Pasadena, CA, United States

**Keywords:** renal cancer, bladder cancer, PD-1, STAT3, antisense oligonucleotides, MDSCs, CpG, TLR9

## Abstract

**Introduction:**

Immune checkpoint blockade (ICB) improved clinical outcomes in renal and bladder cancer patients, but the response rates remain limited especially in metastatic disease. While STAT3 transcription factor is well-known master regulator of tumor immune evasion, little is known about the role of STAT3 in the resistance of renal or bladder cancers to immunotherapy.

**Methods:**

To better understand immune alterations associated with ICB resistance, we assessed blood biomarkers in renal cancer patients classified as responders or non-responders to first line nivolumab/ipilimumab immunotherapy.

**Results:**

We observed that non-responders showed elevated levels of proinflammatory mediators, such as IL-1RA, IL-6, IL-8 and to lesser extent IL-10, which are associated with STAT3 activation and tumor immunosuppression. In addition, we found STAT3 activation primarily in circulating myeloid immune cells such as tolerogenic MDSCs. To assess whether STAT3 inhibition within these cell subsets can promote antitumor immune responses and/or enhance sensitivity to ICB *in vivo*, we used an original antisense oligonucleotide (ASO) strategy for myeloid-cell selective STAT3 knockdown (CpG-STAT3ASO). Our results in syngeneic models of renal and bladder cancers in mice demonstrated potent antitumor activity of CpG-STAT3ASO alone in contrast to PD1 blockade alone in both models. The CpG-STAT3ASO/anti-PD1 combination improved therapeutic efficacy especially against bladder tumors. Therapeutic efficacy correlated with activation of dendritic cells (DCs) and M1 macrophages in the tumor microenvironment, reduced percentages of regulatory T cells (Tregs) and the expansion of CD8 T cells in both tumor models.

**Discussion/Conclusion:**

Our study underscores the potential of using myeloid-cell targeted CpG-STAT3 inhibitors for genitourinary cancer therapy to disrupt tolerogenic signaling, restore immune cell activity and sensitivity to immune checkpoint inhibitors and/or T cell-based immunotherapies.

## Graphical Abstract

CpG-STAT3ASO disrupts tumour-induced immunosuppression by inhibiting activity of STAT3 and stimulating TLR9 signaling in antigen presenting cells, such as DCs ang macrophages. The improved antigen presentation and reduced percentage of immunosuppressive cells such as M2-like macrophages, improves the ratio of CD8 T cells to Tregs and the sensitivity to immune checkpoint blockade as PD1-specific antibodies. Created with BioRender.com. 
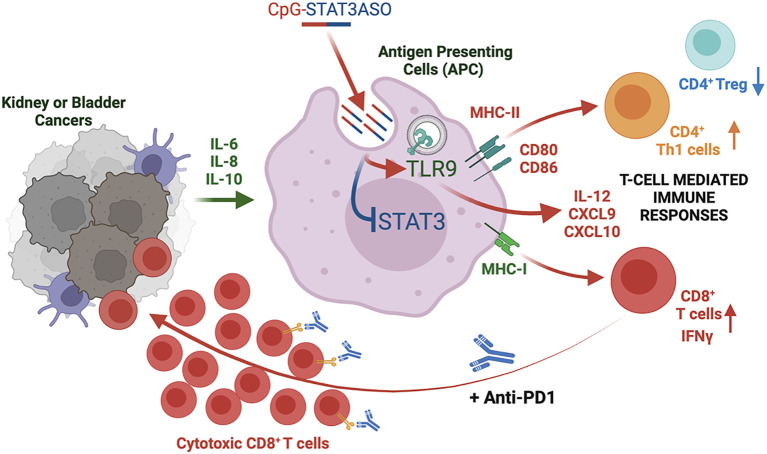

## 1 Introduction

After prostate tumors, renal and bladder cancers are the most common genitourinary malignancies responsible for 2.4% and 2.7% of all cancer deaths in the United States, respectively (1). Unlike in the case of prostate cancers, new immunotherapies based on immune checkpoint blockade (ICB) showed promise for the treatment of both renal cell carcinoma (RCC) and bladder cancer patients (2, 3). FDA-approved ICB strategies for bladder cancers include monoclonal antibodies specific to programmed death-1 (PD-1) or programmed death ligand-1 (PD-L1), while for kidney cancers, PD-1/L1 inhibitors can also be combined with cytotoxic T-lymphocyte-associated protein 4 (CTLA-4)-specific antibodies or small molecule tyrosine kinase receptor inhibitors (3). PD-1 inhibitors improved the objective response rates (ORRs) in metastatic bladder cancer patients to 29% (4), while in RCC patients the combination ICB led to 40% ORRs; however, few patients with metastatic disease achieved durable responses (5, 6). Nonetheless, a large proportion of patients have remained unresponsive to therapy at least partly due to tolerogenic effects of the tumor microenvironment (TME) (3, 7). The immune alterations underlying the resistance of bladder and RCC patients to ICB remain to be fully elucidated, but it is well established that the TME plays an important role in genitourinary cancers and influences the resistance to ICB. In fact, earlier studies from our own group and others interrogating blood and tumor immune markers have suggested that myeloid-derived suppressor cells and tumor-associated macrophages (TAMs) accumulating in RCC patients are associated with poor prognosis (7, 8) with TAMs specifically associated with disease recurrence in clear cell RCC (9, 10). Therefore, myeloid cells remain an attractive but so far challenging target for immunotherapy RCC patients treated with ICB.

Myeloid cells such as dendritic cells, macrophages, and myeloid-derived suppressor cells (MDSCs), as well as Tregs, are key immune cell populations that promote cancer progression by protecting tumors from CD8^+^ T-cell elimination (11). Integral to this landscape are cytokines and chemokines which attract myeloid cells and Tregs and are crucial for inducing an inflammatory cancer-promoting environment. Indeed, circulating cytokines in patient plasma such as interleukin-6 (IL-6), IL-8, and IL-10 were implicated in poor patient responses to ICBs in patients with kidney, breast, and bladder cancer and melanoma (12, 13). All three are known activators of tumorigenic signaling via signal transducer and activator of transcription 3 (STAT3) (14). Both IL-6 and IL-8 have been demonstrated to inhibit antitumor CD8 T-cell responses via recruitment of immunosuppressive myeloid cells or STAT3 activation and arginase-I-mediated suppressive functions of MDSCs in genitourinary and other human cancers (15–18). Indeed, increased STAT3 activation is evident in various cancers including RCC where it is correlated with increased metastasis and poor patient outcomes (19–21). The challenges in targeting STAT3 lie in the pleiotropic STAT3 activity that drives both pro- and antitumor effects. While STAT3 inhibits antigen presentation and promotes the tolerogenic effects of myeloid cells, it is also required for the expansion of cytotoxic CD8 T cells in cancer patients and for the development and maintenance of memory T cells (22, 23). Therefore, small molecule inhibitors of Jak/STAT3 with broad and non-cell selective inhibitory effects can result in conflicting immune effects and prevent long-term antitumor responses. To overcome these limitations, we previously developed a strategy to deliver oligonucleotide-based STAT3 inhibitors, such as antisense oligonucleotides (ASOs), specifically into Toll-like receptor-9 (TLR9) expressing myeloid cells, B cells, and certain cancer cells (24). Conjugation of STAT3ASO to CpG oligonucleotide, a TLR9 agonist, facilitates targeting of TLR9-expressing immune cells, such as human and mouse tumor-associated MDSCs (25) and macrophages (26), plasmacytoid DCs, and B cells but not T cells (27). The cellular selectivity of CpG-STAT3ASO benefits the generation of antitumor immunity by restoring the activity of antigen-presenting cells (negatively impacted by STAT3) without interfering with STAT3 activity in T cells which is required for their expansion and memory T-cell formation (22, 23). Following a rapid internalization within minutes or exposure, CpG-STAT3ASO escapes from endosomes and engages RNase H1 to degrade target mRNAs (24). In our recent studies, systemic administration of CpG-STAT3ASO generated potent immune activity against bone-localized prostate tumors in mice (24, 26).

Thus, we investigated the potential mechanisms contributing to the resistance of RCC patients to combined immune checkpoint blockade (nivolumab/ipilimumab) using blood specimens collected from two recently completed clinical trials at City of Hope. Following these observations, we set out to test the feasibility of using the CpG-STAT3ASO strategy alone and in combination with PD-1 blockade against two models of genitourinary cancers in mice.

## 2 Materials and methods

### 2.1 Patient samples and characteristics

This study examines peripheral blood and plasma samples prospectively collected as part of two clinical trial protocols at City of Hope National Medical Center, in which treatment-naive patients with metastatic renal cell carcinoma treated with nivolumab and ipilimumab were enrolled. Blood samples were collected at baseline and week 12. Response assessment was performed in 12-week intervals by the principal investigator per RECIST 1.1 criteria. Patients with complete or partial response were considered responders, while those with the best response of stable disease or progressive disease were considered non-responders. Both protocols were approved by the City of Hope Institutional Review Board. All study procedures were undertaken in accordance with the Declaration of Helsinki. Demographic and patient responses are shown in **Table 1**.

**Table 1:** Renal cancer patient demographic and responses.

Patient characteristics	Total (*n* = 37)	Non-responders (*n* = 21)	Responders (*n* = 16)
Age (years, average)	64.702 (44–90 years)	63.71 (44–90 years)	66 (45–88 years)
Gender Male Female	30 (81%) 7 (18%)	18 (85%) 3 (14%)	12 (75%) 4 (25%)
IMDC prognostic risk Favorable Intermediate Poor	2 (5%) 28 (75%) 7 (18%)	2 (9.52%) 13 (61.90%) 6 (28.57%)	– 15 (93.75%) 1 (6.25%)
Histologic subtype: Clear cell RCC (#1) Clear cell with sarcomatoid features Papillary RCC (#2) Papillary with sarcomatoid features Sarcomatoid RCC (#3) Poorly differentiated RCC	27 (72.9%) 6 (16.2%) 1 (2.7%) 1 (2.7%) 1 (2.7%) 1 (2.7%)	15 (40.54%) 4 (10.8%) 1 (2.7%) 1 (2.7%)	12 (32.43%) 2 (5.4%) 1 (2.7%) 1 (2.7%)

### 2.2 Quantitative analysis of plasma cytokines/chemokines

Patients who received a first-line combination of nivolumab and ipilimumab in two separate clinical trials at City of Hope were identified, and peripheral blood samples were collected at baseline or at week 12 from 37 RCC patients representing responders (*n* = 16) or non-responders (*n* = 21). We elected to use samples obtained at baseline before treatment initiation and at week 12 ( ± 4 weeks) as typical times used in assessing the initial patients’ immune response. A total of 70 samples obtained from all patients were included in the final analysis. To assess cytokine/chemokine concentration, plasma was separated from peripheral blood mononuclear cells (PBMCs) by centrifugation and stored at −80°C until analysis using a panel of 30 cytokines including IL-1RA, IL-1b, IL-2, IL-2R, IL-4, IL-5, IL-6, IL-7, IL-8, IL-10, IL-12, IL-13, IL-15, IL-17, eotaxin, EGF, FGF, G-CSK, IFN-α, IFN-γ, CXCL9, CXCL10, CCL2, CCL3, CCL4, CCL5, TNF-α, and VEGF on the Luminex Flexmap 3D system (Biotechne, Minneapolis, MN, USA).

### 2.3 Flow cytometric analysis of patients’ immune cells

PBMCs were thawed at 37°C for 5 min, washed in 10% FBS/RPMI 1640, resuspended in the matched patient’s plasma from the same draw (20% plasma/RPMI 1640), and incubated for a minimum of 2 h at 37°C. Following incubation, cells were washed and incubated with a combination of 1 µM of sodium orthovanadate (Sigma-Aldrich, St. Louis, MO, USA) and DNase I (Roche, Basel, Switzerland), before staining with a viability dye and fluorescent antibodies for surface immune markers. Extracellular staining was performed using fluorochrome-labeled antibodies (from BD, Franklin Lakes, NJ unless stated otherwise) to CD3 (#612940), CD19 (#565697), HLA-DR (#565073), CD14 (#563561), CD8 (#612889), PD-1 (#329920), CD4 (# 2500492), CD69 (#562989), CD33 (Thermo-Fisher Scientific, Irwindale, CA, USA, #47033841), PD-L1 (#563742), CD15 (#747426), and CD56 (#565139). For intracellular staining, cells were fixed before permeabilization (Thermo-Fisher Scientific, #00-5523-00) and immunostained for arginase-1 (R&D Systems, Minneapolis, MN, USA, #IC5868F), phosphotyrosine (Y705) STAT3 (#557815), and FoxP3 (#560852).

### 2.4 T-cell proliferation studies

T-cell proliferation studies were performed as described before with minor modifications (24, 26). Briefly, whole blood from patients was cultured for 2 h at 37°C. Next, samples were washed and centrifuged at 250×*g* for 5 min. Cell pellets were then resuspended in red blood cell lysis buffer for 10 min/4°C, treated using DNase for 5 min/37°C, then filtered through a 70-µm nylon mesh filter and washed. CD15^+^ cells were enriched using a positive selection kit from Stemcell Technologies, Vancouver, Canada (#18651) following the manufacturer’s protocol, then cultured in the presence of 20% matched patient’s plasma and 500 nM of CpG-STAT3ASO or control CpG-scrON or with PBS. The following day, CD3^+^ T cells were then enriched using a T-cell enrichment kit (Stemcell Technologies, #17951) from healthy donor PBMCs obtained using density centrifugation over Histopaque-1077 at 1,500 rpm/20 min. T cells were then labeled with CFSE dye according to the manufacturer’s protocols (Thermo-Fisher Scientific, #C34554) and co-cultured with CD15^+^ cells and CD3/CD28 beads in round-bottom 96-well plates (Thermo Fisher, #11131D) at a ratio of 1:6 of T cells to CD15^+^ myeloid cells. After 3 days, flow cytometric analysis was performed to assess T-cell proliferation using CFSE dilution using antibodies specific to CD8a (RPA-T8, #25-0088-42).

### 2.5 Oligonucleotide design and synthesis

The CpG oligonucleotide conjugates were synthesized in the DNA/RNA Synthesis Core (COH) as previously described (24). The resulting oligonucleotide (ON) conjugates are shown below (x = a single C3 unit; underline = 2′O-methyl-modification; asterisk = phosphorothioation site):

CpG(D19)-human STAT3ASO:

5′ G*G*TGCATCGATGCAG*G*G*G*G*G-xxxxx-C*A*G*C*A*G*A*T*C*A*A*G*T*C*C*A*G*G*G*A 3′.

STAT3 ASO (human STAT3 ASO targeting sequence):

5′ C*A*G*C*A*G*A*T*C*A*A*G*T*C*C*A*G*G*G*A 3′.

CpG(D19)-scrambled oligonucleotide (scrON):

5′ G*G*TGCATCGATGCAG*G*G*G*G*G-xxxxx-A*G*A*G*C*C*T*A*A*C*G*G*A*A*G*G*C*A*C*T 3′.

CpG (1668)-mouse STAT3ASO:

5′ T*C*C*A*T*G*A*C*G*T*T*C*C*T*G*A*T*G*C*T-xxxxx-G*A*C*T*C*T*T*G*C*A*G*G*A*A*T*C*G*G*C*T 3′.

CpG (1668)-scrambled oligonucleotide (scrON):

5′ T*C*C*A*T*G*A*C*G*T*T*C*C*T*G*A*T*G*C*T-xxxxx-A*G*A*G*C*C*T*A*A*C*G*G*A*A*G*G*C*A*C*T* 3′.

### 2.6 Mouse tumor models and animal studies

Mouse kidney (Renca) and bladder (MB49) cancer cells were purchased from the American Type Culture Collection (ATCC). Renca and MB49 cells were cultured in RPMI 1640 or DMEM media, respectively, supplemented with 10% FBS, 1% penicillin/streptomycin, and 1% GlutaMAX (Thermo-Fisher). All cell lines were cultured for less than 3 months prior to the experiments and were tested to be free of *Mycoplasma* infection.

Balb/C and C57BL/6 mice, aged between 6 and 8 weeks, were purchased from the Jackson Laboratory. Mouse care and experimental conditions were performed under pathogen-free conditions and in accordance with established institutional guidance and approved protocols from the Institutional Animal Care and Use Committees. For efficacy studies with the subcutaneously (SC) implanted Renca tumors, 5 × 10^5^ Renca cells were resuspended in a 1:1 ratio with Matrigel (Corning, Corning, NY, USA, #356231) and 1× PBS and injected SC into female Balb/C mice. Mice were then treated intraperitoneally (IP) with the anti-PD-1 antibody on day 3 and day 5 (200 µg, BioXCell, Lebanon, NH, USA, #BE0273) before treatment every other day with CpG-STAT3ASO (5 mg/kg) in combination with anti-PD-1, or alternatively, mice were treated with CpG-STAT3ASO (5 mg/kg) alone or IgG or PBS control, all injected intravenously (IV) via retro-orbital venous sinus injection. Tumor size was monitored and measured every other day using calipers. For the SC MB49 mouse model, 5 × 10^5^ MB49 cells were resuspended in 1× PBS and injected SC into male C57BL/6 mice. When tumors reached approximately 100 mm^3^, mice were treated twice using IP injections of anti-PD-1 antibody (200 µg) (BioXCell, Cat #BE0273) 2 days apart, before treatment 2 days later with CpG-STAT3ASO (5 mg/kg) in combination with anti-PD-1, or alternatively, mice were treated with CpG-STAT3ASO (5 mg/kg) alone or IgG or PBS control injected IV. In the animal study using subcutaneous administration of oligonucleotides, mice were first treated IP with anti-PD-1 antibody on day 3 and day 5 (200 µg, BioXCell, #BE0273) before treatment on days 7, 9, 11, 13, and 15 with or without SC-injected CpG-STAT3ASO (10 mg/kg) on days 7, 9, 11, 13, and 15.

For the analysis of tumor-associated immune cells, single-cell suspensions were prepared from whole tumors or tumor-draining lymph nodes using short collagenase-IV/DNase-I treatment (20 min/37°C) followed by mechanical dispersion by pipetting through a 70-µm mesh filter. Viable cells were enriched using density centrifugation over Histopaque-1083 at 1,500 rpm/20 min. Extracellular staining was performed with fluorochrome-labeled antibodies (from BD unless stated otherwise) to CD45 (#564279), CD86 (#741285), CD11c (#612797), CD80 (#562611), CD11b (Thermo Fisher, #48-0112-82), CD8 (#563063), CD206 (#141721), Gr1 (BioLegend, San Diego, CA, USA, #50-604878), MHC II (Thermo Fisher, #11-5321-82), CD3 (#560527), CD4 (R&D Systems, #FAB554S-100), and FoxP3 (#566881). Fluorescence data were analyzed on BD Fortessa LSR II and the Cytek Aurora Spectral Cytometer and using FlowJo v10 software (TreeStar, Ashland, OR, USA) or Cytobank (Beckman-Coulter, Brea, CA).

### 2.7 Circulating IFNγ and IL-6 levels in the peripheral blood of Renca and MB49 mice

Peripheral blood samples were obtained from tumor-bearing mice using tail vein bleed on day 14. Blood was centrifuged at 1,200 rpm/5 min to separate plasma which was then stored at −80°C until use. IFNγ and IL-6 concentrations in plasma were assessed using ELISA assays (Thermo Fisher, #KMC4021 or #KMC0061, respectively) according to the manufacturer’s instructions.

### 2.8 Statistical analysis

For the patients’ sample analysis and animal studies, comparisons of groups were performed using the Wilcoxon signed-rank test, one-way ANOVA, or two-way ANOVA with Bonferroni’s multiple comparisons test. Data were presented as mean ± SEM. Statistical significance was ranked and indicated as follows: *, *p* < 0.05; **, *p* < 0.01; ***, *p* < 0.001; ****, *p* < 0.0001.

## 3 Results

### 3.1 Elevated levels of immunoregulatory mediators in renal cancer patients’ refractory to first-line nivolumab/ipilimumab therapy

**Figure 1:**
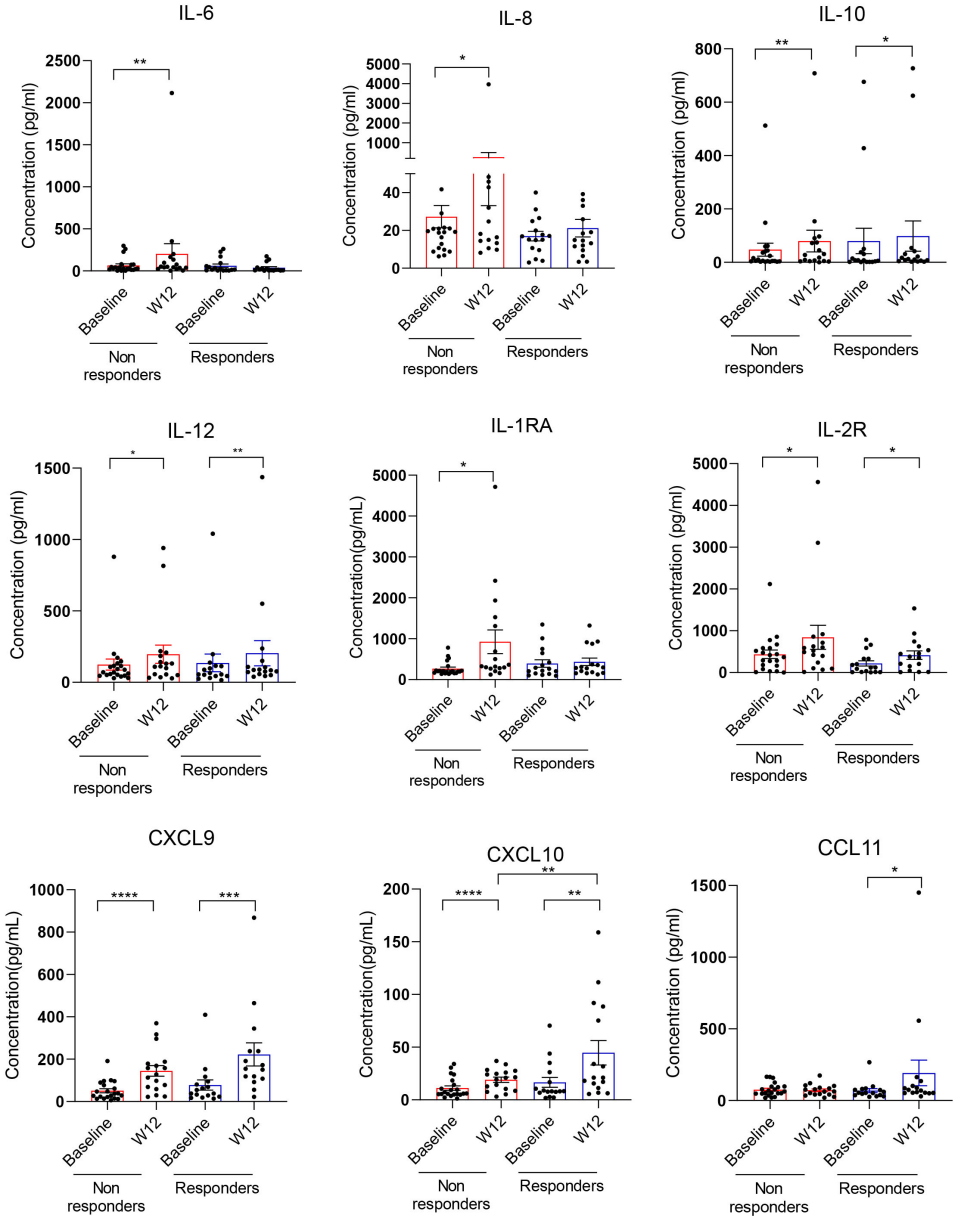
Plasma cytokine and chemokine analysis in renal cell carcinoma (RCC) patients responding or non-responding to combined immune checkpoint blockade (ICB) therapy. Plasma samples from 37 mRCC patients who received a combination of nivolumab and ipilimumab as first-line therapy were collected at baseline and week 12. Response to therapy was assessed using RECIST 1.1 criteria (please see **Table 1**). Thirty cytokines/chemokines were measured in patient plasma between baseline and week 12 and assessed in responders (*n* = 16) or non-responders (*n* = 21) using Luminex assays. Shown are means ± SEM. Statistical significance was determined by the Wilcoxon signed-rank test; only statistically significant differences were indicated: *p < 0.05, **p < 0.01, ***p < 0.001, ****p < 0.0001.

To assess the effects of combination immune checkpoint therapy (ICB) on plasma immune mediators, peripheral blood was obtained from all patients at baseline and at week 12 when the first indications of response to clinical immunotherapies often occur. We assessed immune alterations in 30 plasma cytokines and chemokines in samples from a total of 37 patients (**Figure 1** and **Supplementary Figure S1**). As shown in **Figure 1**, the refractory patients/non-responders showed a significant increase in immunoregulatory cytokines such as IL-6, IL-8, IL-10, and IL-1RA. In contrast, only IL-10 was weakly upregulated in responding patients. IL-6, IL-8, and IL-10 are important STAT3 activators and are often associated with the recruitment or expansion of tolerogenic MDSCs (14, 28, 29). Protein markers of ongoing immune response such as IL-12, soluble IL-2R, and CXCL9 and CXCL10 were significantly elevated after combined ICB therapy in both groups of patients. However, the levels of IFN-inducible CXCL10 were significantly higher in responding than in non-responding patients after 12 weeks of ICB therapy, which is likely indicative of IFN-driven immune responses critical for long-term antitumor effects. We also observed a significant increase in proinflammatory, innate immune regulator, CCL11/eotaxin, in some of the responding patients.

### 3.2 STAT3 activation in circulating myeloid immune cells in RCC patients

**Figure 2:**
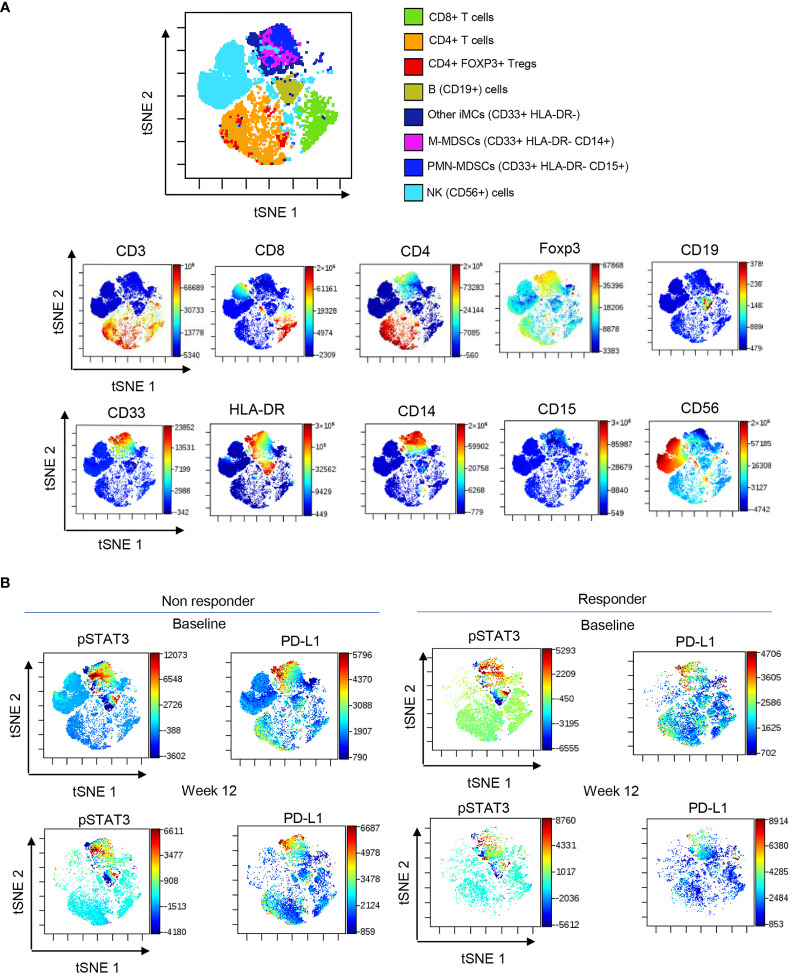
STAT3 activity in circulating immune cell subsets in ICB-treated RCC patients. PBMCs were collected from RCC patients at baseline and week 12 and analyzed using spectral flow cytometry. **(A)** viSNE/t-SNE map overlay showing immune cell clusters allocated by an unsupervised analysis using the dimensionality reduction algorithm (top) and the expression pattern of markers associated with each cluster (bottom). **(B)** Levels of pSTAT3 and PD-L1 expression in different cell population clusters in responders and non-responders between baseline and week 12.

**Figure 3:**
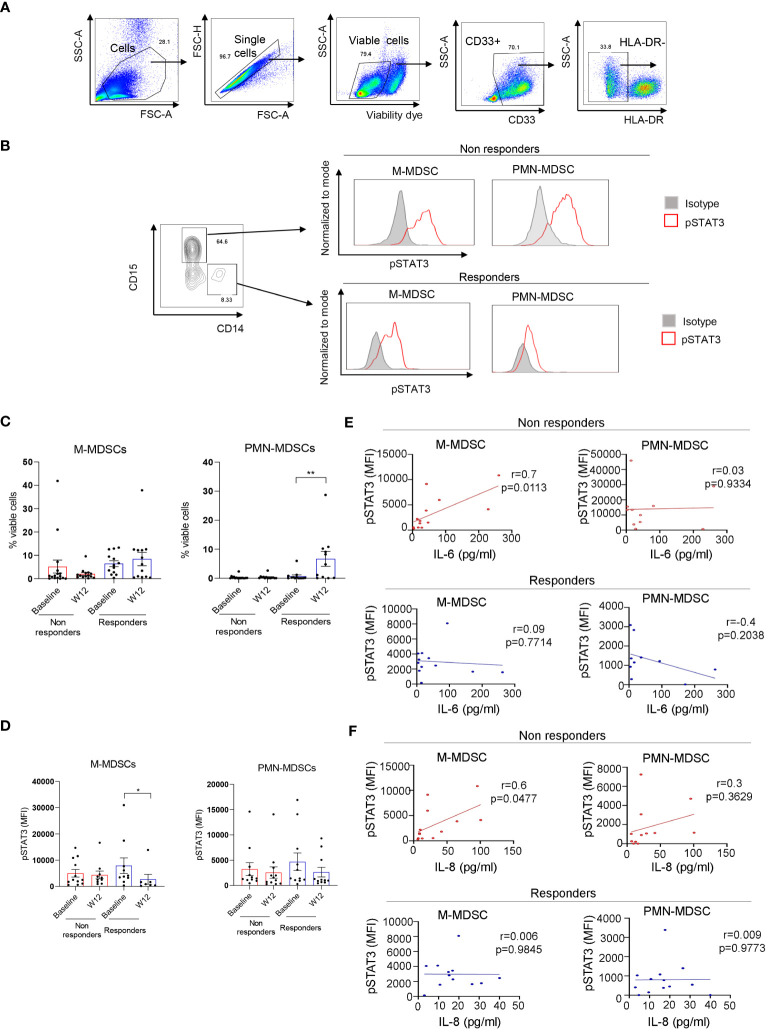
STAT3 is activated in myeloid-derived suppressor cell (MDSC) populations in mRCC patients’ blood. Patients’ blood samples obtained at baseline and week 12 were analyzed using flow cytometry. **(A)** Gating strategy used to assess polymorphonuclear MDSCs (PMN-MDSCs) (CD33^+^CD15^+^HLA-DR^−^) and M-MDSCs (CD33^+^CD14^+^HLA-DR^−^). **(B)** pSTAT3 levels in both MDSC populations in a representative baseline sample. **(C, D)** Total percentages of M-MDSCs and PMN-MDSCs **(C)** and the levels of STAT3 activity in both MDSC subsets **(D)** were assessed in the blood from non-responders (*n* = 16) and responders (*n* = 13) at baseline and week 12. Shown are the means ± SEM. **(E, F)** Correlations between baseline levels of STAT3 activation in M-MDSCs and PMN-MDSCs and plasma levels of IL-6 **(E)** or IL-8 **(F)** as measured by flow cytometry in non-responders (*n* = 13, red) and responders (*n* = 10, blue) as measured using Luminex. Shown are the means ± SEM. Statistical significance was determined by the Wilcoxon signed-rank test with the SEM test. Pearson coefficient (*r*) and *p*-values are shown for each correlation. *p < 0.05, **p < 0.01.

STAT3 is commonly activated in the microenvironment of human tumors including RCC (30). Given the elevated plasma levels of several STAT3 activators, we assessed STAT3 phosphorylation across circulating immune cell populations using high-parameter spectral flow cytometry. As shown in **Figure 2**, t-distributed stochastic neighbor embedding (t-SNE), a non-linear dimensionality reduction algorithm, indicated STAT3 activation (pSTAT3) and the expression of STAT3 downstream target and immune checkpoint molecule PD-L1 in overlapping myeloid cell clusters, likely polymorphonuclear MDSCs (PMN-MDSC: CD15^+^CD33^+^HLA-DR^−^) and monocytic MDSCs (M-MDSCs: CD14^+^CD33^+^HLA-DR^−^) at the treatment initiation as well as after 12 weeks of ICB therapy. We also detected modest STAT3 within a cluster of circulating B cells; however, the percentages of B cells did not significantly change during the ICB therapy and did not show a positive correlation with STAT3-inducing cytokines (**Supplementary Figure S2**). The analysis of both MDSC subsets confirmed the upregulation of pSTAT3 at baseline in both responders and non-responders (**Figures 3A, B**, gating strategy). While there was no significant change in the total percentage of M-MDSCs before and after ICB therapy, a subset of patients responding to therapy showed an increase in the percentage of PMN-MDSCs (**Figure 3C**). Importantly, responding but not the refractory patients showed a significant reduction in the activity of STAT3 in M-MDSCs between baseline and week 12. However, we did observe a decrease in pSTAT3 in M-MDSCs in patients who responded to therapy between baseline and W12 (**Figure 3D**). Correspondingly, we observed a strong correlation between the level of STAT3 activity in M-MDSCs and plasma concentrations of IL-6 (**Figure 3E**; *r* = 0.7, *p* = 0.0113) as well as IL-8 (**Figure 3F**; *r* = 0.6, *p* = 0.0477) in non-responding patients. We did not find such a correlation between pSTAT3 and plasma cytokine levels in patients responding to therapy or in PMN-MDSCs (**Figures 3E, F**). Our observations suggest that nivolumab/ipilimumab immunotherapy has a partial effect on the tolerogenic myeloid cells accumulating in RCC patients, especially on the subset of M-MDSCs. However, the presence of both MDSC populations after 12 weeks of therapy in both non-responding and responding patients suggests that STAT3-driven immunosuppression may limit the clinical efficacy of combined immune checkpoint inhibition. In fact, we have not observed an increase in the percentages of CD8^+^ T-cell subsets in ICB-treated RCC patients. Instead, there was a modest but significant increase in the percentage of regulatory T cells (Tregs) specifically in non-responders but not in responding patients (**Supplementary Figure S3**).

### 3.3 Targeting RCC patients’ MDSCs using CpG-STAT3ASO restores T-cell activity

**Figure 4:**
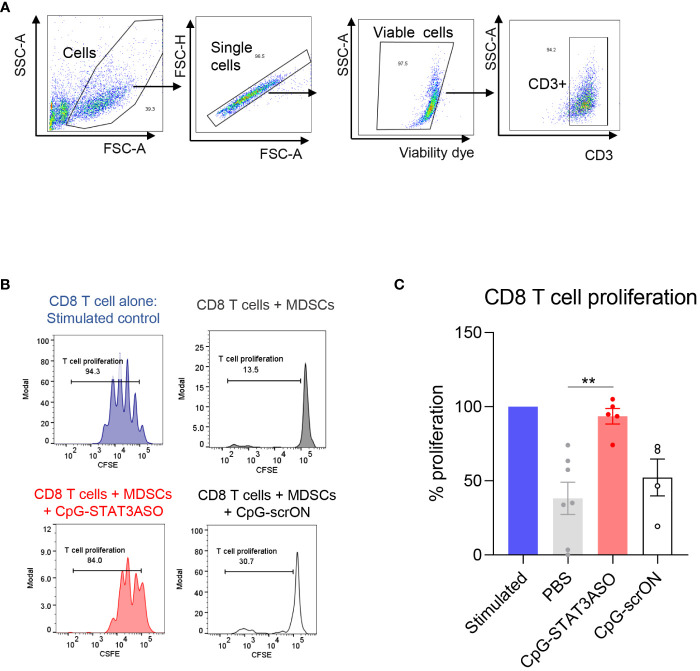
CpG-STAT3ASO restores T-cell proliferation in the presence of mRCC patient-derived MDSCs. CD15^+^ PMN-MDSCs were enriched from RCC patients’ PBMCs and treated with 500 nM CpG-STAT3ASO, control CpG-scrON, or PBS for 24 (h) Then, MDSCs were co-cultured with healthy donor T cells for 3 days before assessing T-cell proliferation using the CFSE dilution assay. **(A)** Gating strategy for T-cell populations. **(B)** CD8 T-cell proliferation in the presence of PMN-MDSCs is restored by CpG-STAT3ASO. Shown are the representative results from one of two independent experiments. **(C)** Shown are the means ± SEM (*n* = 5–7/experimental group); **p < 0.01.

The persistent presence of tolerogenic MDSCs in the blood of nivolumab/ipilimumab-treated RCC patients suggested a potential mechanism of STAT3-driven therapeutic resistance. Targeting STAT3 signaling in RCC-derived MDSCs could alleviate their immunosuppressive effects and restore T-cell proliferation and activity. To test this hypothesis, we used a myeloid cell-selective CpG-STAT3ASO strategy. CpG-STAT3ASO is selectively internalized by TLR9^+^ myeloid cells, including human MDSCs but not by T lymphocytes (24). Primary CD15^+^ myeloid cells, including PMN-MDSCs, were enriched from treatment-refractory RCC patients’ PBMCs and incubated with CpG-STAT3ASO to knockdown STAT3 or with non-targeting but immunostimulatory CpG-scrON oligonucleotide (**Figures 4A, B**). Next, we co-cultured the treated MDSCs with healthy donor T cells for 3 days and assessed T-cell proliferation using the flow cytometric assay. As shown in **Figure 4C**, CpG-STAT3ASO but not control CpG-scrON almost completely abrogated the inhibitory effect of RCC-derived MDSCs on T-cell proliferation. The control CpG-scrON, lacking STAT3 inhibitory activity but activating TLR9, had only limited and non-significant stimulatory effect on T-cell proliferation (*p* = 0.008). These results imply that CpG/TLR9 stimulation alone without STAT3 inhibition is not sufficient for disrupting the tolerogenic effects of RCC-associated myeloid cells.

### 3.4 Systemic administration of CpG-STAT3ASO alone and in combination with PD-1 blockade shows efficacy against kidney and bladder tumors in mice

**Figure 5:**
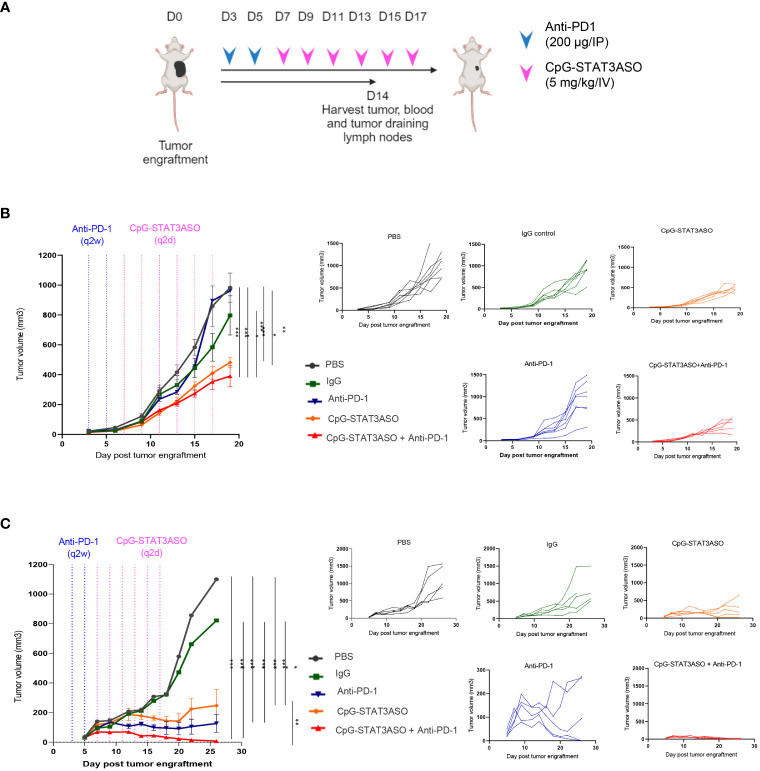
Systemic administration of CpG-STAT3ASO alone and in combination with anti-PD-1 shows efficacy against kidney and bladder tumors in mice. **(A)** Study outline and treatment regimens for the efficacy studies. **(B, C)** Mice were engrafted subcutaneously with either Renca kidney tumors **(B)** or MB49 bladder tumors **(C)** and treated using two IP injections of anti-PD-1 or control IgG (200 µg/injection) and/or six IV injections of CpG-STAT3ASO (5 mg/kg) or PBS. Tumor growth kinetics was monitored using caliper measurements. Shown are the representative results as combined data (left panel) or individual growth curves per treatment group (five right panels) from two independent experiments for each model; mean ± SEM (*n* = 5). Statistical significance was determined with two-way ANOVA with Bonferroni’s multiple comparisons test; *p < 0.05, **p < 0.01, ***p < 0.001, ****p < 0.001.

Next, we set out to assess whether STAT3 inhibition/TLR9 stimulation using CpG-STAT3ASO will prove effective alone or together with immune checkpoint blockade against genitourinary cancers, such as Renca and MB49 tumors, two commonly used models of mouse kidney and bladder cancers (**Figure 5A**). We first tested the effect of systemic administration of CpG-STAT3ASO with or without prior PD-1 blockade on the growth of Renca tumors. As shown in **Figure 5B**, repeated IV injections of CpG-STAT3ASO alone significantly reduced kidney tumor progression in comparison to all control groups. Our control study (**Supplementary Figure S4A**) and the published results in prostate tumor models (24) confirmed that concomitant TLR9 activation and STAT3 inhibition are necessary for the generation of effective antitumor effects. In contrast, Renca tumors were completely resistant to PD-1 blockade alone, even after four more intensive treatments (**Supplementary Figure S4B**), while the combination of anti-PD-1 with CpG-STAT3ASO showed modest improvement in the antitumor effect (**Figure 5B**). We also confirmed that the subcutaneous route of CpG-STAT3ASO delivery, at a site distant from the tumor, also resulted in an antitumor effect, suggesting an alternative systemic route of administration for this oligonucleotide (**Supplementary Figure S4C**).

We next assessed the efficacy of CpG-STAT3ASO and anti-PD-1 as single treatments and the combination in the MB49 bladder cancer model (**Figure 5C**). Compared with the Renca model, CpG-STAT3ASO resulted in a more pronounced antitumor effect against MB49 tumors with significant growth inhibition and occasional complete tumor eradication. PD-1 blockade alone demonstrated significant although less consistent oligonucleotide treatment antitumor efficacy against MB49 tumors. Finally, the combination of CpG-STAT3ASO and anti-PD-1 resulted in augmented antitumor efficacy leading to complete tumor regression in the majority of mice. These results suggested potential differences in the cellular mechanisms of antitumor effects likely related to the different compositions of the tumor microenvironment in both kidney and bladder cancer models.

### 3.5 TLR9-targeted STAT3 inhibition alone reactivates antigen-presenting myeloid cells in the microenvironment of kidney and bladder tumors

**Figure 6:**
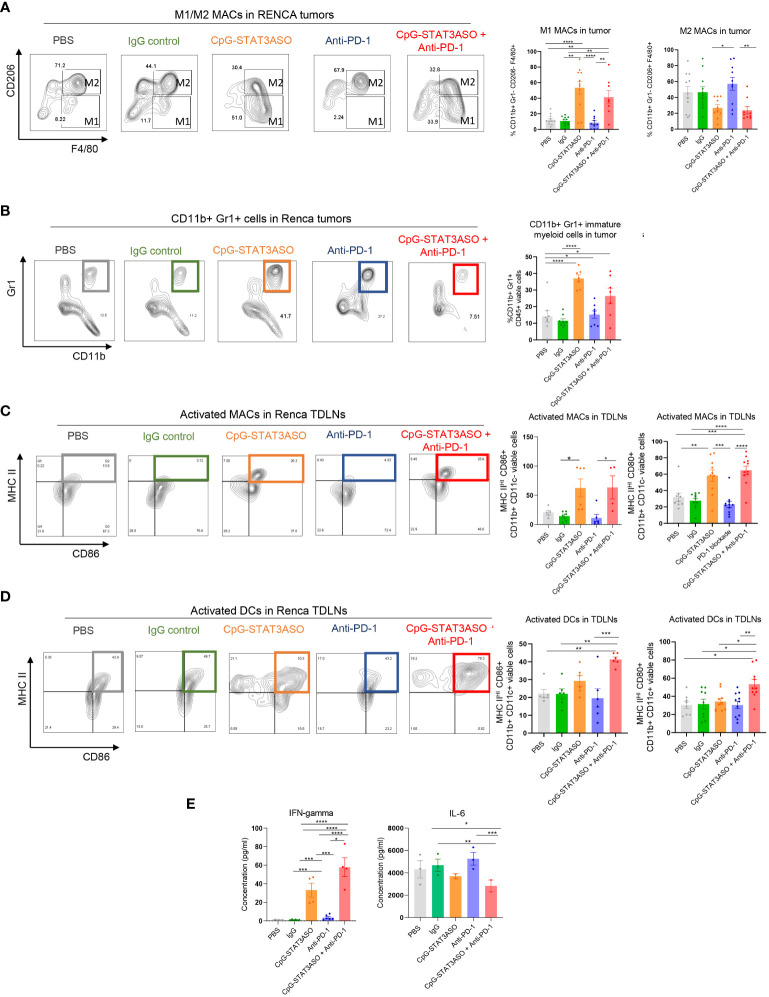
Characterization of the immunostimulatory effects of CpG-STAT3ASO and/or anti-PD-1 on the Renca kidney tumor microenvironment. Mice engrafted subcutaneously with Renca tumors were treated as described in **Figure 5A**. Tumors and tumor-draining lymph nodes were harvested for flow cytometric analysis. **(A)** CpG-STAT3ASO but not anti-PD-1 alone results in an M2- to M1-like phenotype shift in the population of Renca tumor-associated macrophages. Representative plots (five left panels) and bar graphs representing the combined results with the percentages of M1- or M2-like macrophage subsets. **(B)** CpG-STAT3ASO alone promotes the recruitment of CD11b^+^Gr1^+^ cells, likely representing neutrophils. Representative plots (five left panels) and bar graphs representing the combined results. **(C)** CpG-STAT3ASO results in the accumulation of activated M1-like macrophages expressing antigen-presenting MHC-II complexes and CD80 and CD86 costimulatory molecules in tumor-draining lymph nodes. **(D)** The combination of CpG-STAT3ASO with PD-1 inhibition, but not either treatment alone, drives the recruitment of activated DCs into tumor-draining lymph nodes. **(E)** Plasma levels of IFNγ and IL-6 in Renca tumor-bearing mice after various treatments as assessed using ELISA. For all results, shown are the means ± SEM (*n* = 5–10). Shown are the results representative of three independent experiments. Statistical significance was assessed with one-way ANOVA with Bonferroni’s multiple comparisons, *p < 0.05, **p < 0.01, ***p < 0.001, ****p < 0.0001.

To assess cellular mechanisms of the observed antitumor effects against Renca and MB49 tumors, we characterized the immunophenotype of the major tumor-associated myeloid cell populations, such as macrophages and DCs. As shown in **Figure 6A** (with gating strategy in **Supplementary Figure S6**), both CpG-STAT3ASO and CpG-STAT3ASO/anti-PD-1 combination treatment dramatically increased the percentage of M1-like macrophages in Renca tumors, four- to five-fold higher than in anti-PD-1 or control treatment groups. In addition, both CpG-STAT3ASO and CpG-STAT3ASO/anti-PD-1 treatments seemed to reduce the percentage of tumor-associated M2-like macrophages compared with PD-1 blockade alone, although this effect did not reach significance in relation to negative controls (**Figure 6A** and **Supplementary Figure S7**). This is consistent with the direct effect of PD immune blockade on T cells and not myeloid cells. Our previous studies using genetic STAT3 deletion in tumor-infiltrating myeloid cells or using CpG-STAT3ASO in prostate cancer models suggested that TLR9 activation/STAT3 inhibition results in the recruitment of CD11b^+^Gr1^+^ myeloid cells representing not MDSCs but neutrophils, which can contribute to antitumor effects (24, 31). In fact, CpG-STAT3ASO alone and, to a lesser extent, the combination treatment elevated the percentage of CD11b^+^Gr1^+^ cells in Renca tumors (**Figure 6B**). Corresponding to these potential immunostimulatory effects at the tumor site, we observed strong recruitment of activated and antigen-presenting (MHC-II^HI^/CD86^+^ or MHC-II^HI^/CD80^+^) M1-like macrophages into Renca tumor-draining lymph nodes by CpG-STAT3ASO and the combination treatment resulting in three- to four-fold increase of the activated macrophages compared with PBS- or antibody-treated controls (**Figure 6C**). We also assessed the recruitment of activated DCs given their important role in antigen presentation and T-cell-mediated antitumor immunity. Interestingly, unlike in the case of macrophages, the combination CpG-STAT3ASO/anti-PD-1 treatment but not CpG-STAT3ASO or anti-PD-1 alone led to a significant recruitment of activated (MHC-II^HI^ and CD86^+^ or CD80^+^) DCs into tumor-draining lymph nodes (**Figure 6D** and **Supplementary Figure S5**). This effect suggested a potentially indirect role of PD-1 blockade in DC activation through IFNγ-dependent mechanism as recently suggested by others (32). In fact, the plasma levels of IFNγ were significantly higher in Renca tumor-bearing mice after the combination CpG-STAT3ASO/anti-PD-1 treatment compared with CpG-STAT3ASO or PD-1 blockade alone with the latter showing only a minor effect. In contrast, the plasma levels of IL-6 were decreased by both CpG-STAT3ASO and the combination treatment but not by PD-1 inhibition alone (**Figure 6E**) and correlated with the previously observed decrease of tolerogenic M2-like macrophages, which are the potential sources of this cytokine in the TME (**Figure 6A**).

**Figure 7:**
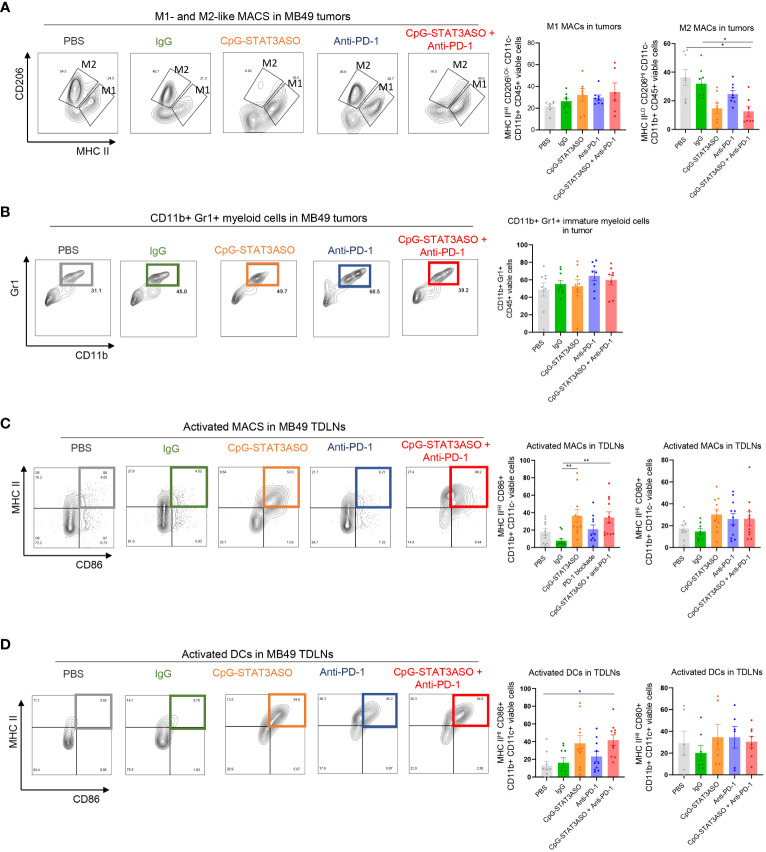
Characterization of the immunostimulatory effects of CpG-STAT3ASO and/or anti-PD-1 on the MB49 bladder tumor microenvironment. Mice engrafted subcutaneously with MB49 tumors were treated as described in **Figure 5A**. Tumors and tumor-draining lymph nodes were harvested for flow cytometric analysis. **(A)** CpG-STAT3ASO but not anti-PD-1 alone results in the reduction of M2-like (CD206^HI^MHC-II^LO/−^) macrophages in the tumor microenvironment with a little increase in M1-like macrophage subset (CD206^LO/−^MHC-II^HI^). Representative plots (five left panels) and bar graphs representing the combined results with the percentages of M1- or M2-like macrophages. **(B)** Tested treatments do not affect the percentages of tumor-infiltrating CD11b^+^Gr1^+^ cells. Representative plots (five left panels) and bar graphs representing the combined results. **(C, D)** Modest recruitment of activated MHC-II^+^/CD86^+^ macrophages **(C)** and DCs **(D)** into tumor-draining lymph nodes by CpG-STAT3ASO and/or combination treatments. For all results, shown are the means ± SEM (*n* = 5–10). Shown are the results representative of three independent experiments. Statistical significance was assessed with one-way ANOVA with Bonferroni’s multiple comparisons, *p < 0.05, **p < 0.01, ***p < 0.001.

As typical for human bladder cancers, the mouse MB49 tumor microenvironment is dominated by Th2 cytokines, such as IL-10, which leads to the accumulation of tolerogenic macrophages as well as regulatory T cells (31, 33). Thus, we expected potential differences in the antitumor effect of the tested CpG-STAT3ASO and PD-1 inhibition between Renca and MB49 models. Nonetheless, similar to kidney tumors, we did observe a significant reduction of CD206^HI^ M2-like macrophages by CpG-STAT3ASO/anti-PD-1 combination in comparison to PBS- and IgG-treated groups, with a less robust inhibitory effect of CpG-STAT3ASO alone (**Figure 7A**). In contrast to the Renca model, there was only a minimal increase in the number of CD11b^+^CD11c^–^MHCII^+^ M1-like macrophages (**Figure 7A**) or CD11b^+^Gr1^+^ cells (**Figure 7B**). However, the combined CpG-STAT3ASO/anti-PD-1 treatment and to a lesser extent CpG-STAT3ASO alone strongly increased the recruitment of activated, antigen-presenting MHC-II^HI^/CD86^+^ macrophages (**Figure 7C**) and DCs (**Figure 7D** and **Supplementary Figure S5**) into tumor-draining lymph nodes. Overall, the results of our immunophenotypic analysis of Renca and MB49 tumor microenvironments correspond well with the differences in antitumor efficacy of various treatments and confirm that CpG-STAT3ASO/anti-PD-1 combination immunotherapy in both tumor types results in more consistent and robust immune stimulation of the tumor-associated myeloid cells.

### 3.6 The combined CpG-STAT3ASO/anti-PD-1 immunotherapy activates CD8 T cells in kidney and bladder tumor models by different mechanisms

**Figure 8:**
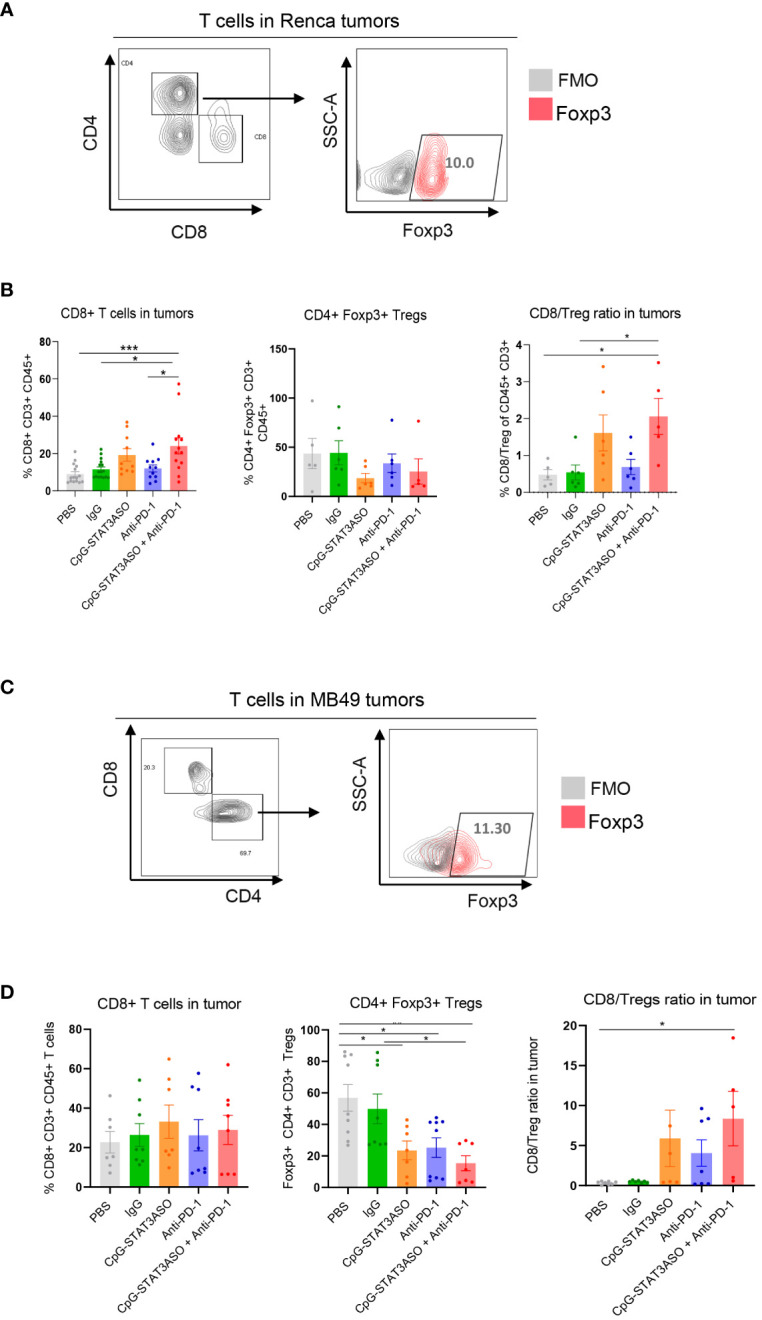
The combined CpG-STAT3ASO/anti-PD-1 immunotherapy activates CD8 T cells in kidney and bladder tumor models by different mechanisms. Mice engrafted subcutaneously with Renca **(A, B)** or MB49 **(C, D)** tumors were treated as described in **Figure 5A**. **(A, C)** Gating strategies for the immunophenotyping of T lymphocytes. **(B)** CpG-STAT3ASO/PD-1 combination and to a lesser extent CpG-STAT3ASO alone increase the recruitment of CD8 T cells into Renca tumors with a less pronounced reduction in CD4^+^/FoxP3^+^ Treg numbers, thereby improving the CD8/Treg ratio. **(D)** CpG-STAT3ASO/PD-1 and CpG-STAT3ASO have little effect on CD8 T-cell recruitment but significantly reduce the percentage of CD4^+^/FoxP3^+^ Tregs. For all the results, shown are the means ± SEM (*n* = 5–6). Statistical significance was assessed with one-way ANOVA with Bonferroni’s multiple comparisons, *p < 0.05, **p < 0.01, ***p < 0.001.

Given the different myeloid cell activation by CpG-STAT3ASO and/or anti-PD-1 treatments, we next assessed the effect on T-cell populations in both tumors. As shown in **Figures 8A, B**, we found a small population of tumor-infiltrating CD8 (~10% of total T cells on average) together with CD4 helper and regulatory CD4^+^/FoxP3^+^ T cells (<50% of total CD4^+^ T cells) in Renca tumors. The combined CpG-STAT3ASO/anti-PD-1 treatment significantly increased the percentage of tumor-infiltrating CD8 compared with PBS and IgG controls (**Figure 8A**). It also showed a tendency to reduce Treg numbers from an average of 50% to 25% although without reaching statistical significance (*p* > 0.05). Thus, CpG-STAT3ASO/anti-PD-1 augmented the CD8/Treg ratio indicative of successful adaptive, antitumor immunity. While CpG-STAT3ASO showed a similar tendency to recruit CD8 T cells to Renca tumors, its overall effect was less robust and not significant given the limited number of tested animals. In contrast to kidney tumors, MB49 bladder cancers were infiltrated by a significant number of tumor-resident CD8 T cells (>20% of total CD3^+^ T cells on average), and regulatory T cells were dominating the CD4 T-cell population (>50% of CD4 T cells) (**Figures 8C, D**). Both the combined CpG-STAT3ASO/anti-PD-1 treatment and CpG-STAT3ASO alone dramatically reduced the percentage of Tregs in MB49 tumors to 15% from an average of 56% in PBS controls. Even though neither of the treatments affected the overall numbers of tumor-resident CD8 T cells, the reduction in the number of Tregs benefitted the ratio of CD8 to Treg cells, thus enabling the generation of antitumor immune responses. The effect of myeloid cell-selective CpG-STAT3ASO on Tregs was likely indirect through disruption of the immunosuppressive myeloid tumor microenvironment that is required for sustaining Treg expansion and activity. Finally, unlike in the Renca tumor model, PD-1 inhibition alone had modestly but significantly reduced the percentage of Tregs improving the CD8 to Treg ratio. This unexpected anti-PD-1 activity may explain its antitumor activity in some of the mice bearing bladder tumors and lack of any effect against the renal cancer model.

## 4 Discussion

Previous studies in RCC patients suggest that tumor-associated myeloid cells play an important role in ICB resistance (34, 35). However, the impact and role of myeloid cells in ICB resistance are not fully understood and have only been assessed in terms of their presence and/or abundance within tumors. In addition, the impact of myeloid cells has not been assessed in patients treated with a combination of nivolumab and ipilimumab. In this study, we assessed immune alterations in patients refractory to combination ICBs nivolumab and ipilimumab and observed alterations in patients associated with myeloid cells, specifically MDSCs, and STAT3. Then, to further support this observation, in RCC mouse models, we also show that PD-1 blockade alone is inefficient at overcoming myeloid-specific alterations evident in RCC tumors. While we were unable to investigate tumor-associated macrophages in these patients, we have extended these studies to animal tumor models of kidney and bladder cancers. Results from both of these models support our hypothesis that a combination of T-cell and myeloid cell targeting allows to overcome immunosuppression and to initiate antitumor immune responses.

In both Renca and MB49 tumor models, CD11b^+^Gr1^+^ cells represent a heterogeneous group of immature myeloid cells, MDSCs, specifically after CpG-STAT3ASO treatment, and neutrophils with antitumor activity (24). Due to the difficulty in defining this population functionally, we focused our studies on the syngeneic tumor models on better-defined macrophage subsets. One of the main mediators and drivers of myeloid cell recruitment and STAT3 activation are cytokines, cytokine receptors, and growth factor receptors (18). Indeed, increased IL-8 in plasma and tumors of RCC patients has been correlated with reduced responsiveness to PD-L1 therapy, and here, we similarly observed increased circulating IL-8 in RCC patients who are non-responders to nivolumab/ipilimumab combination. In addition, we also observed increased IL-6 in non-responders to the combined ICB, which suggests activation of STAT3 in non-responders. Combined with the observation of the predominant M2-like macrophage phenotype in anti-PD-1-alone-treated Renca kidney tumors, this suggests a potential role of TAMs in promoting resistance of kidney tumors to ICB therapy. At the same time, the relatively small number of available RCC patients suggests the need for further validation of our results in future studies in larger cohorts of patients.

Macrophages have a demonstrated capacity to suppress immune responses via nutrient depletion, recruitment of immunosuppressive T regulatory cells, and direct suppression of T-cell function (36). Indeed, in our studies, we observed that patient-derived RCC myeloid cells have the ability to suppress T-cell function, and in Renca models, tumors of PBS- and IgG control-treated RCC mice, a large proportion of macrophages exhibited an M2 phenotype characterized by CD206^+^ expression. In comparison, in tumors of CpG-STAT3ASO alone and in the combination PD-1 blockade and CpG-STAT3ASO-treated RCC mice, we observed an increase of F4/80^+^ or M1-like phenotype macrophages commonly associated with classical activation or antitumor responses (37). Interestingly, treatment with PD-1 blockade alone had no observed effect on TAMs and exhibited similar results to PBS and IgG antibody control-treated groups in the RCC (Renca) model. This suggests firstly that myeloid cells, specifically macrophages, play a role in ICB resistance and that combined CpG-STAT3ASO and anti-PD-1-mediated reprogramming of macrophages promotes antitumor immunity resulting in increased tumor growth control and a concurrent increase in CD8^+^ T-cell infiltration and CD8/Treg ratios in combination-treated tumors. Intriguingly, in Renca tumors, the CpG-STAT3ASO/anti-PD-1 combination had a superior effect on the activation of DCs. This observation suggests that PD-1 blockade had an indirect role in DC activation likely through the mechanism involving IFNγ-mediated stimulation of IL-12 production by DCs as recently reported (32). Alternatively, the improved DC activation may also result from the enhanced immunogenic cell death of cancer cells after the combined treatment. The release of damage-associated molecular patterns (e.g., mitochondrial DNA) provides additional stimuli for innate immune receptors such as TLR9 or STING/cGAS (27). Overall, our data underscore the benefit of combining myeloid cell-targeted CpG-STAT3ASO strategy with PD-1^+^ T-cell-directed immune checkpoint blockade. At the same time, it is important to note that the absence of a complete antitumor response in the RCC (Renca) model points out that further improvements of the antitumor immunity may require combinations with other PD-1 or CTLA4 immune checkpoint inhibitors, for example, TIM-3 or LAG3, which are commonly expressed in human RCC (38, 39). Beyond the effects on myeloid cell populations, we previously showed that CpG-STAT3ASO can reduce the numbers of intratumoral Tregs in prostate tumor models in mice as well as in the xenotransplanted head and neck tumors in humanized mice (23, 40). Given that CpG-STAT3ASO is not internalized by T cells, the effect of this oligonucleotide on the population of Tregs is likely indirect and likely driven by the disruption of the immunosuppressive network of myeloid cells with the reduction of STAT3 activators and Th2-/Treg-promoting cytokines such as IL-6 and IL-10 (31, 41).

Our current findings underscore the potential of using myeloid cell-targeted STAT3 inhibition to overcome resistance of genitourinary cancers, such as kidney and bladder cancers, to immunotherapy and trigger CD8 T-cell-mediated antitumor immune responses. Both CpG ONs and STAT3ASO molecules were well tolerated by patients when tested as single agents in clinical trials (42, 43). The ongoing IND-enabling safety and toxicokinetic studies of CpG-STAT3ASO will serve as the basis for a planned phase I clinical trial in patients with genitourinary cancers. We believe that this study can provide a rationale for the use of myeloid cell-selective inhibitors of STAT3 signaling alone and in combination with the already approved immune checkpoint inhibitors in order to augment therapeutic efficacy and durability of responses in patients with GU cancers and potentially also other solid tumors.

## Data availability statement

The raw data supporting the conclusions of this article will be made available by the authors, without undue reservation.

## Ethics statement

The studies involving humans were approved by City of Hope National Medical Center IRB committee. The studies were conducted in accordance with the local legislation and institutional requirements. The participants provided their written informed consent to participate in this study.

## Author contributions

MA: Conceptualization, Funding acquisition, Investigation, Software, Writing – original draft, Writing – review & editing, Data curation, Formal analysis, Methodology, Project administration, Resources, Validation, Visualization. WT: Investigation, Methodology, Writing – review & editing. DW: Investigation, Writing – review & editing, Methodology. DK: Writing – review & editing, Data curation. EK: Writing – review & editing, Data curation. ND: Conceptualization, Project administration, Resources, Writing – review & editing. AC-R: Methodology, Project administration, Resources, Writing – review & editing. LM: Methodology, Project administration, Resources, Writing – review & editing. ZZ: Methodology, Project administration, Resources, Writing – review & editing. JHa: Writing – review & editing. JHs: Writing – review & editing, Project administration. CE: Methodology, Writing – review & editing. DM: Methodology, Resources, Writing – review & editing, Data curation, Project administration. AH: Writing – review & editing, Methodology, Resources, Funding acquisition. SP: Conceptualization, Investigation, Methodology, Project administration, Resources, Supervision, Writing – review & editing. MK: Conceptualization, Data curation, Formal analysis, Funding acquisition, Investigation, Methodology, Project administration, Resources, Supervision, Validation, Writing – original draft, Writing – review & editing.
