# Supplementary figures and images for: Targeting STAT3 in tumor-associated antigen-presenting cells as a strategy for kidney and bladder cancer immunotherapy
Source: Front Immunol. 2024 Jan 8;14:1274781. doi: 10.3389/fimmu.2023.1274781 (PMC10800835; doi:10.3389/fimmu.2023.1274781)

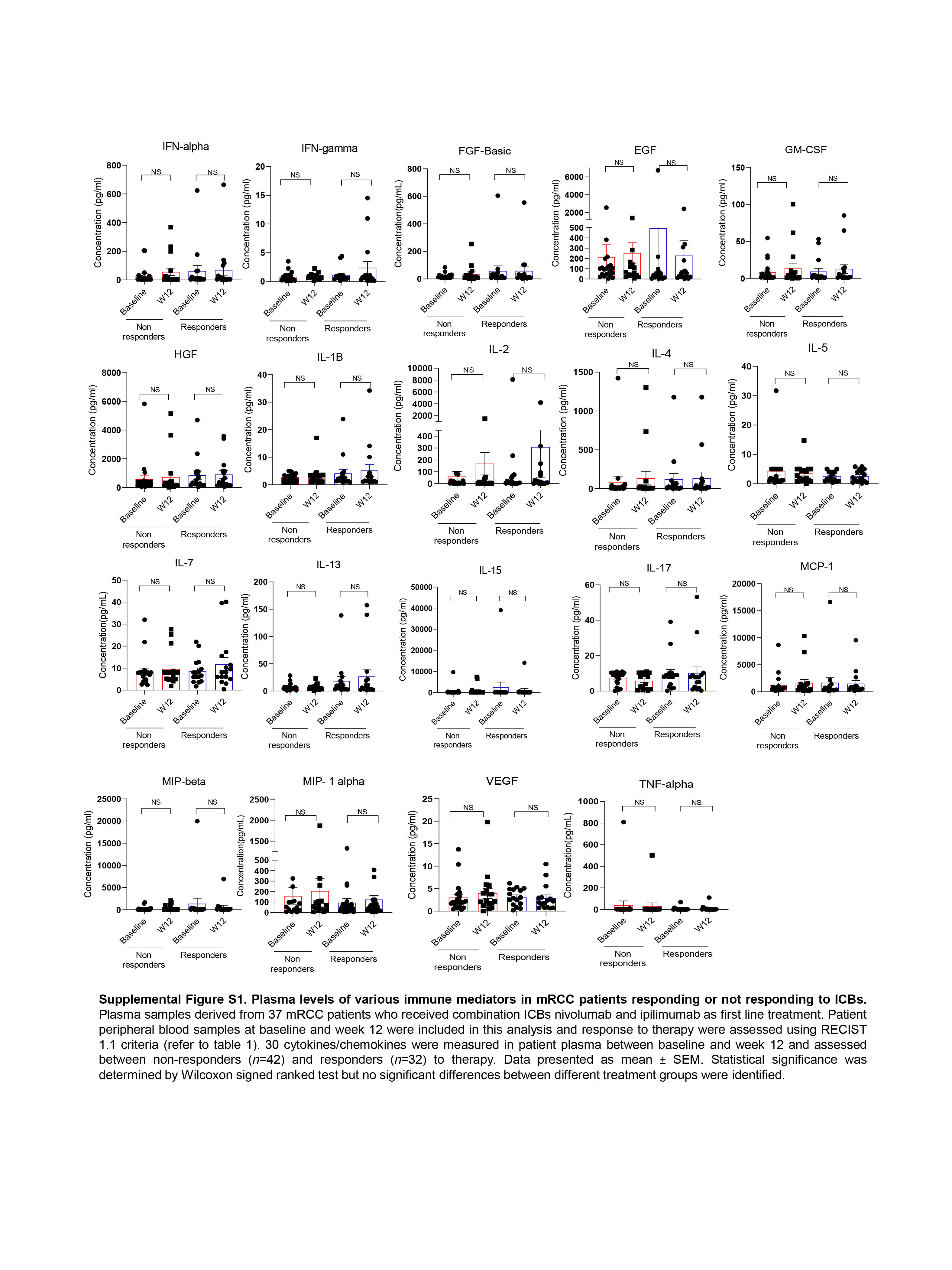

Supplement: Supplementary file 1 [file Image_1.tif]

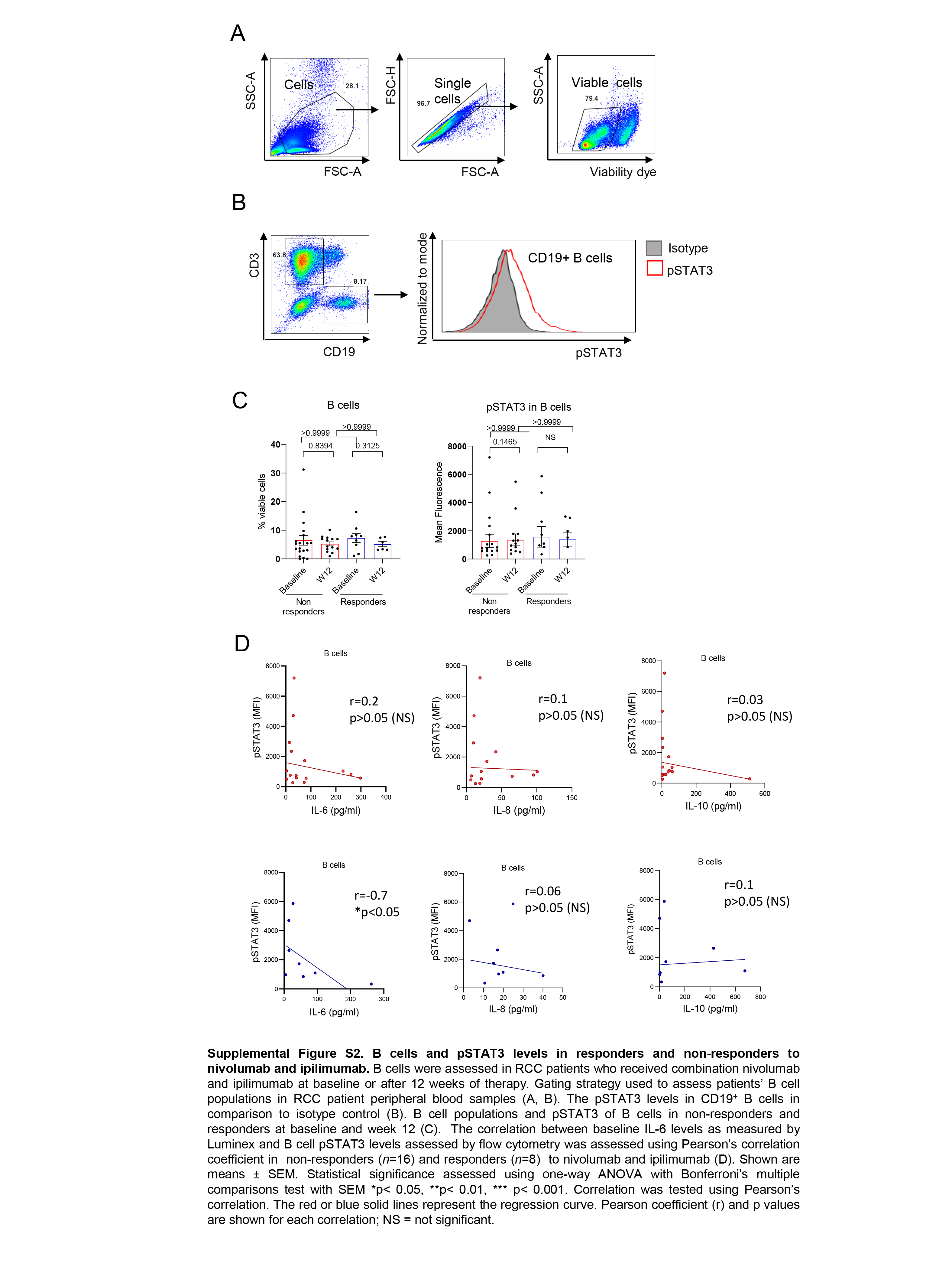

Supplement: Supplementary file 2 [file Image_2.tif]

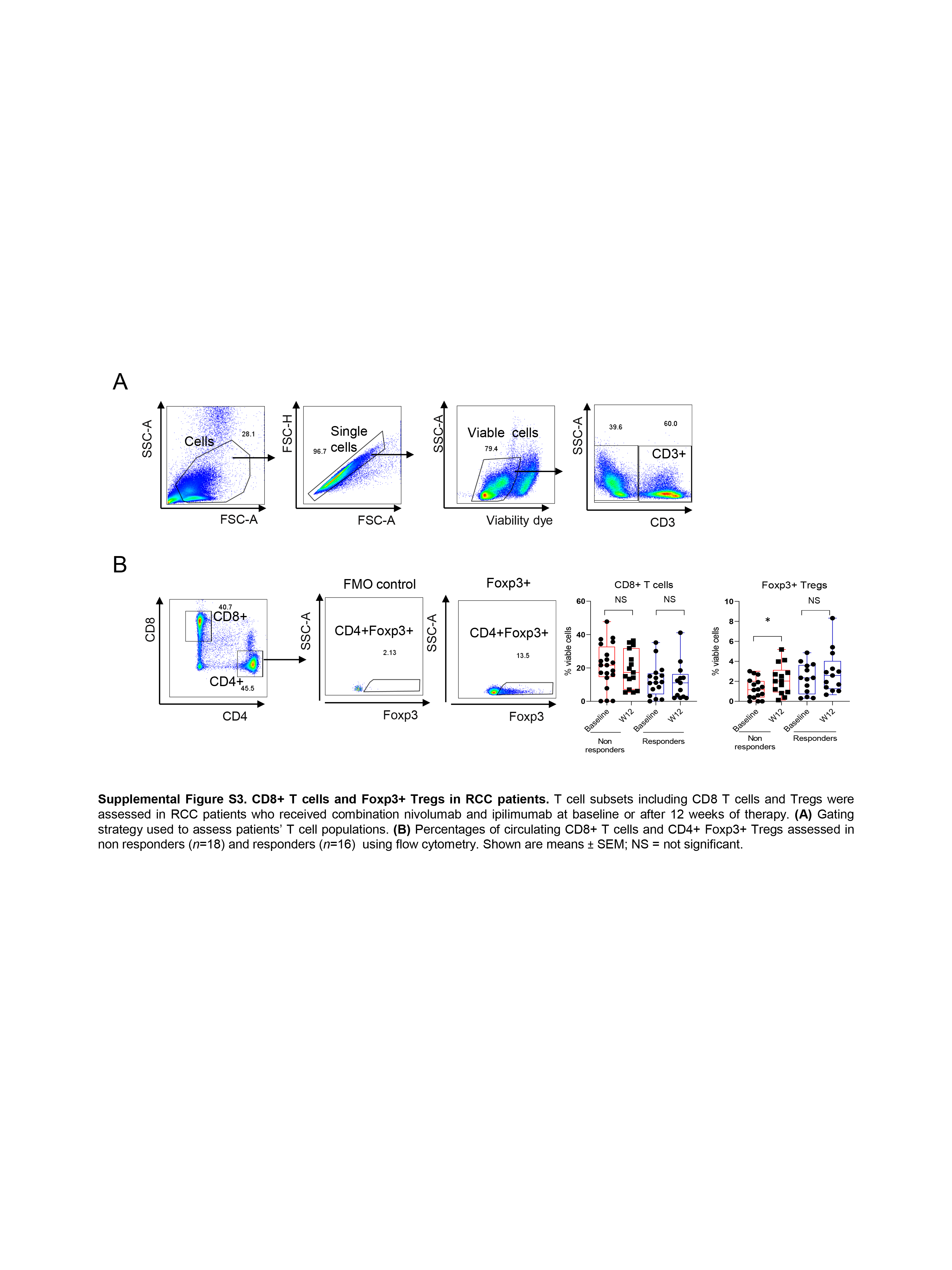

Supplement: Supplementary file 3 [file Image_3.tif]

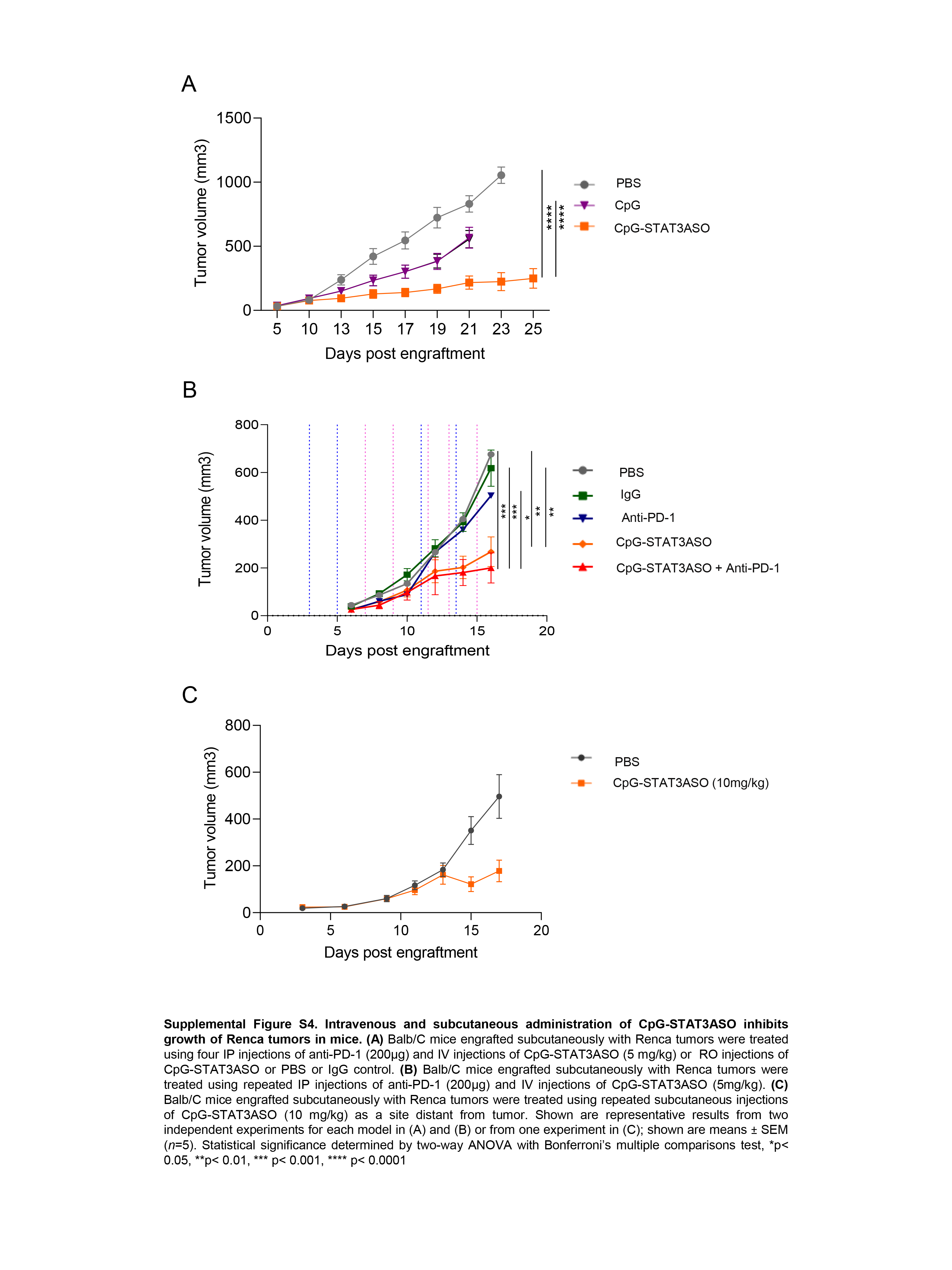

Supplement: Supplementary file 4 [file Image_4.tif]

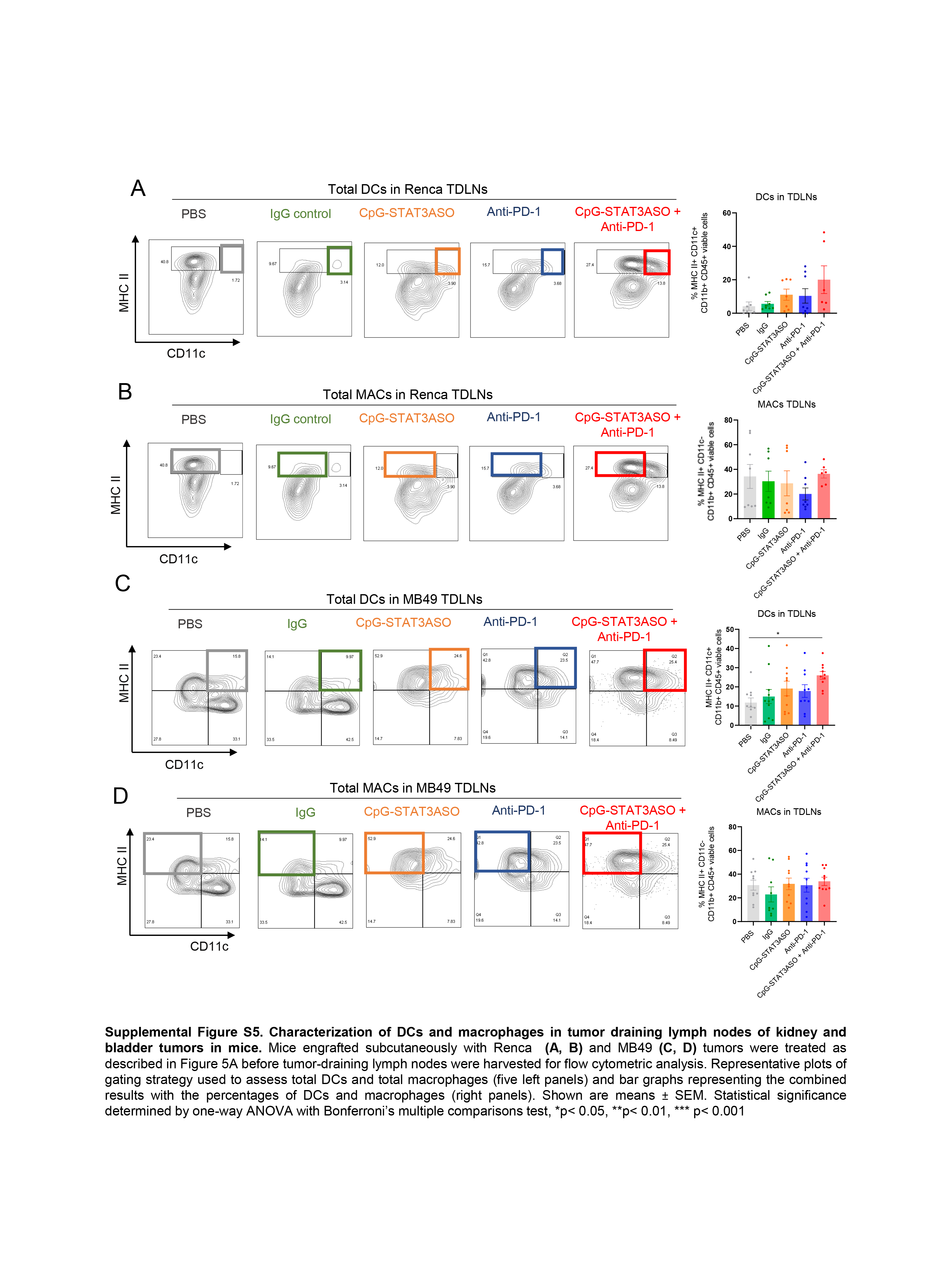

Supplement: Supplementary file 5 [file Image_5.tif]

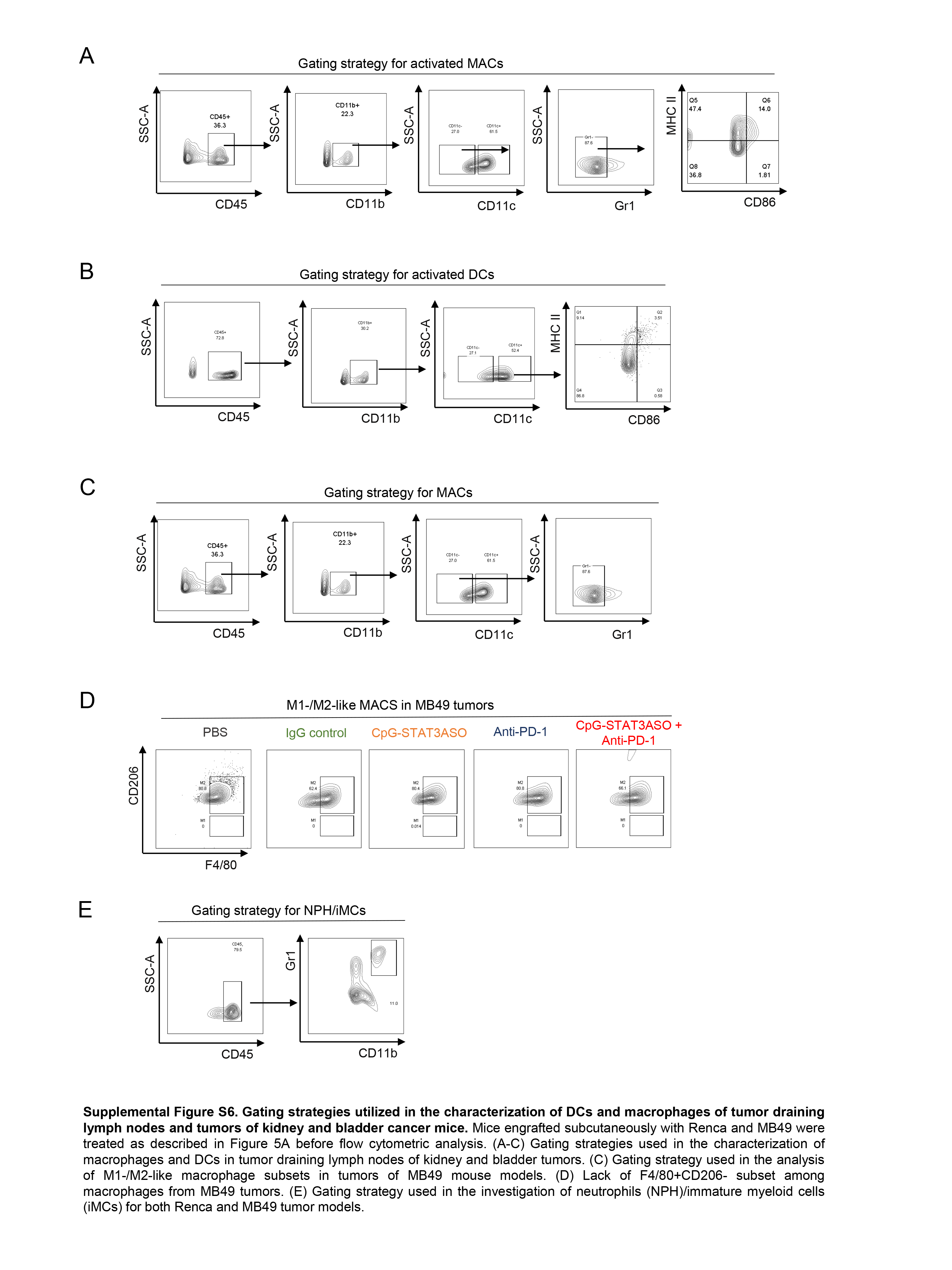

Supplement: Supplementary file 6 [file Image_6.tif]

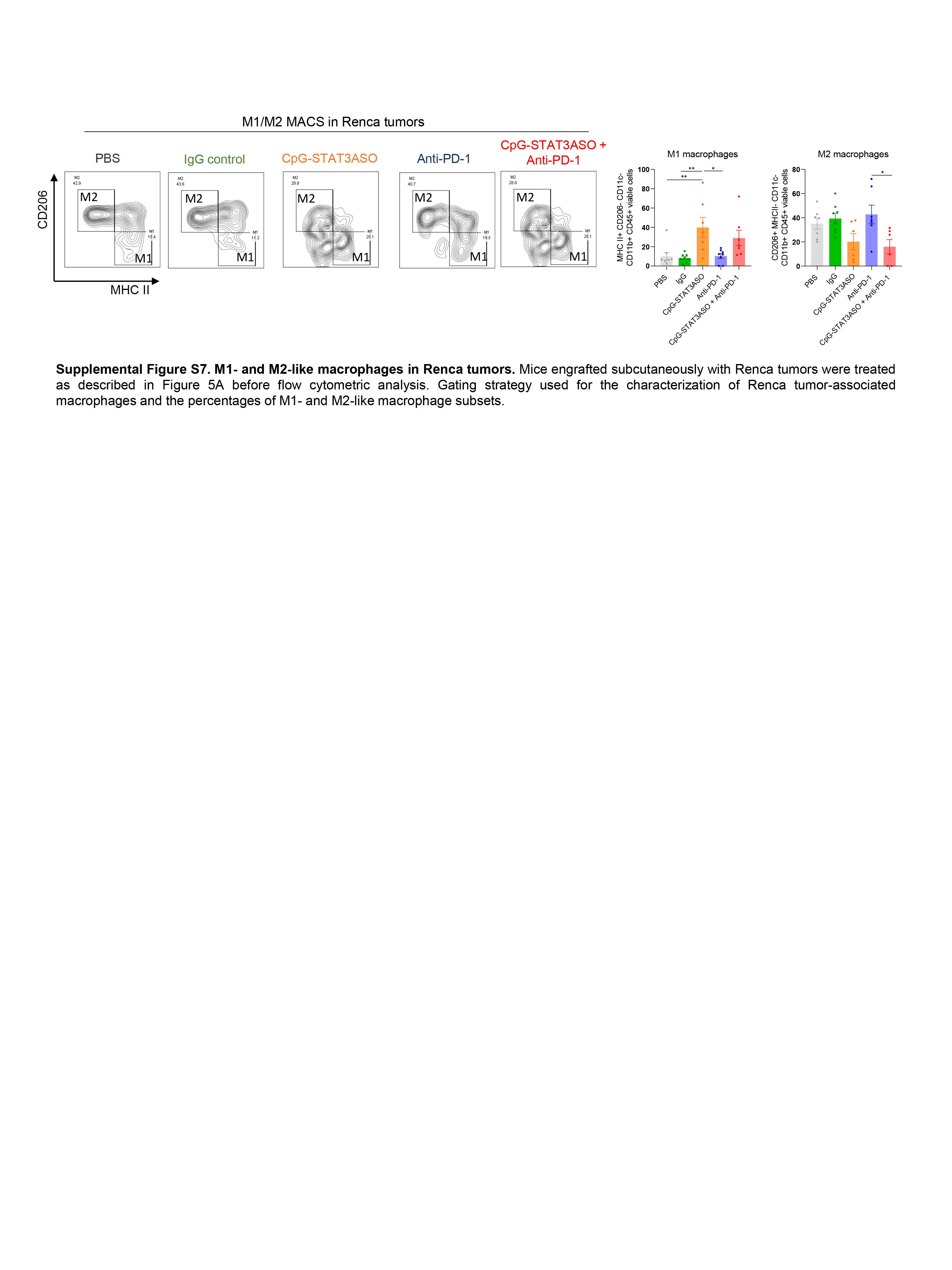

Supplement: Supplementary file 7 [file Image_7.tif]
